# Metastasis of cervical cancer indicated by elevation of serum CA125 produced by mediastinal lymph nodes: a case report

**DOI:** 10.1186/s13256-024-04417-2

**Published:** 2024-02-25

**Authors:** Ken-ichi Honda, Masato Miyama, Yuko Nishii, Reiko Tasaka, Yusuke Nakano, Naohiko Umesaki, Takeshi Fukuda, Tomoyuki Ichimura, Tomoyo Yasui, Toshiyuki Sumi

**Affiliations:** 1 Department of Obstetrics and Gynecology, PL Hospital, 2204 Shindou, Tondabayashi, Osaka 584-8585 Japan; 2 Department of Gynecology, Izumi City General Hospital, Izumi, Japan; 3 MedCity21, Osaka, Japan; 4 Department of Obstetrics and Gynecology, Graduate School of Medicine, Osaka Metropolitan University, Osaka, Japan; 5 Department of Oncology, Izumi City General Hospital, Izumi, Japan

**Keywords:** Cervical cancer, Biomarker, SCCA, CA125, Lymph nodes

## Abstract

**Background:**

In patient assessment for recurrence of neoplasia, a biomarker that shows an elevated serum value before the first treatment is a candidate for follow-up examination. The biomarker squamous cell carcinoma antigen is usually utilized for follow-up of squamous cell cancer of the cervix.

**Case presentation:**

We herein report a 30-year-old Japanese woman of postoperative metastasis of cervical squamous cell cancer to the mediastinal and supraclavicular lymph nodes as indicated by an elevated serum cancer antigen 125 concentration and not by the squamous cell carcinoma antigen value. After chemoradiotherapy and chemotherapy, the serum cancer antigen 125 concentration decreased to a normal value. Squamous cell carcinoma antigen was found to be distributed in both the squamous cell cancer tissue of the cervix and the supraclavicular lymph node metastatic tissue. By contrast, cancer antigen 125 was distributed in the supraclavicular lymph node metastatic tissue but not in the original squamous cell cancer tissue of the cervix.

**Conclusion:**

In this case, metastasis of cervical cancer to the mediastinal and supraclavicular lymph nodes was shown by the biomarker cancer antigen 125, which was not present in the original neoplasia.

## Background

Squamous cell carcinoma of the cervix is usually assessed with the biomarker squamous cell carcinoma antigen (SCCA), which reflects the tumor status [1, 2]. We encountered a patient in whom metastasis of cervical squamous cell carcinoma to the lymph nodes was not reflected by the serum SCCA value but instead by an elevated serum cancer antigen 125 (CA125) value.

CA125 was historically identified by a monoclonal antibody against an ovarian cancer cell line (OVCA433) [3]. CA125 is a cell membrane-spanning glycoprotein that serves as a biomarker for ovarian cancer and other tumors of female reproductive organs. Interaction of the CA125 epitope with the mesothelial lining may help tumor cells bind to the peritoneal cell membrane [4, 5]. Previous reports have described cases of lung cancer and pleural diseases characterized by an elevated serum CA125 concentration [6]. Our experience suggests there are common factors between female reproductive organs and mediastinal lymph nodes, which may be related to the biological roles of CA125.

## Case presentation

**Fig. 1:**
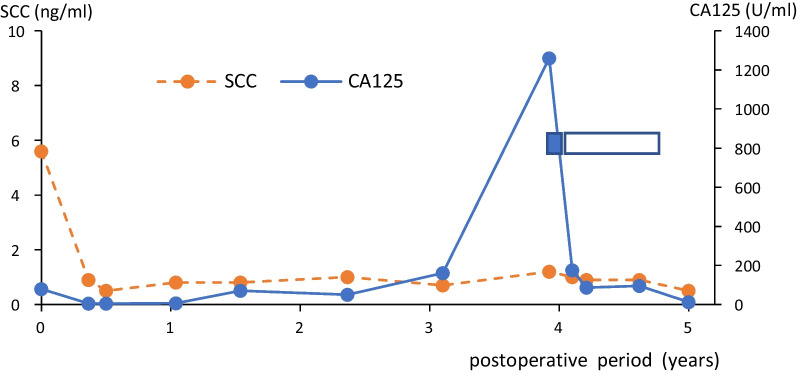
Serum levels of SCCA and CA125. The blue bar indicates the period of chemoradiotherapy, and the white bar indicates the period of chemotherapy. SCCA, squamous cell carcinoma antigen; CA125, cancer antigen 125

**Fig. 2:**
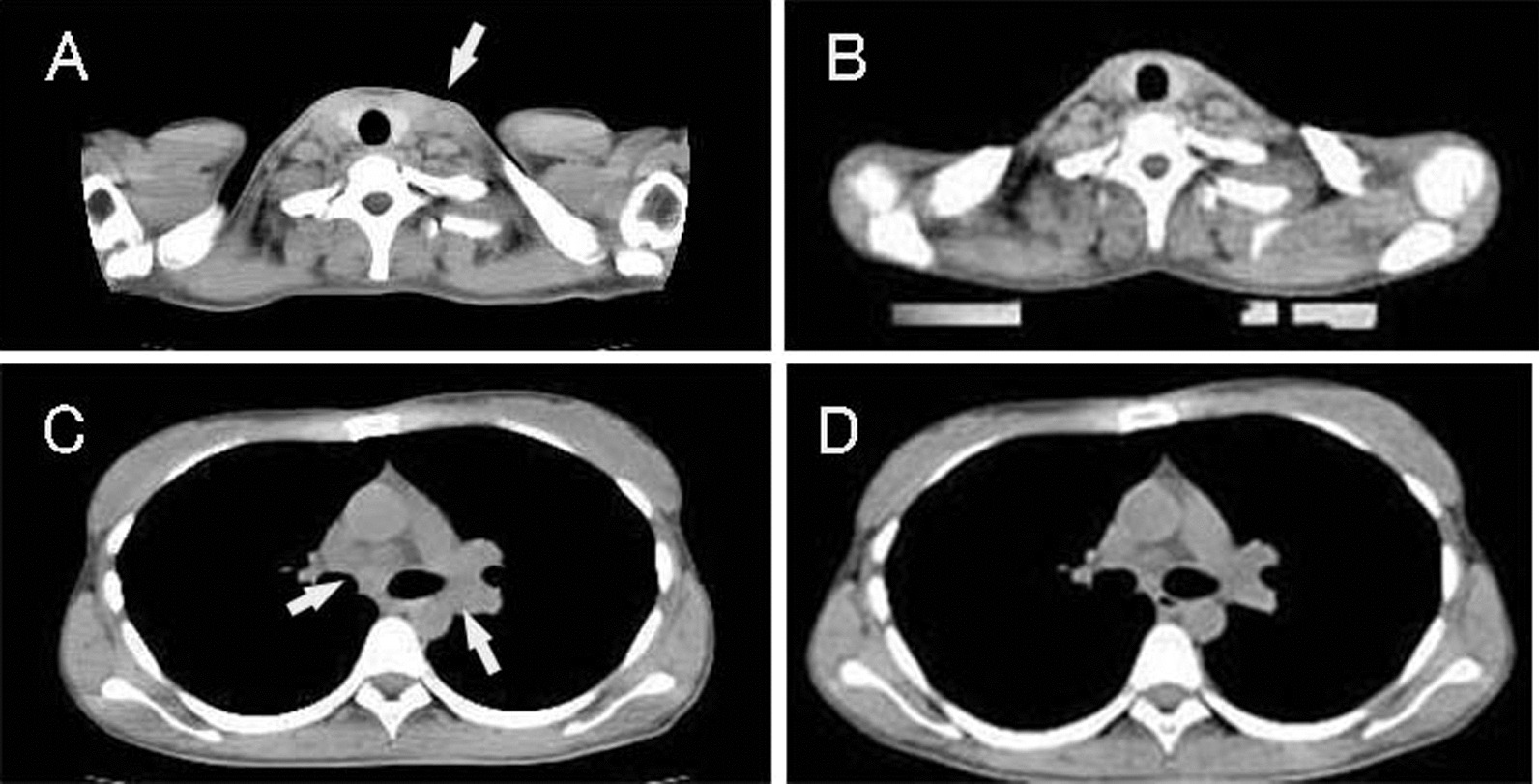
Computed tomography images. **A**, **B** Supraclavicular area and **C**, **D** mediastinal area. **A**, **C** Before and **B**, **D** after chemoradiotherapy. The arrow in **A** indicates enlarged left supraclavicular lymph nodes, and the arrows in **C** indicate enlarged bilateral hilar lymph nodes

A 27-year-old Japanese woman (gravida 0) underwent radical hysterectomy and left adnexectomy for stage 1b cervical cancer and an endometrial cyst of the left ovary, followed by four cycles of postoperative adjuvant chemotherapy with intravenous paclitaxel and carboplatin drips at Izumi City Hospital (the previous institution of Izumi General Hospital). Her serum level of squamous cell carcinoma antigen (SCCA) was 5.6 ng/mL before radical hysterectomy and 0.9 ng/mL after postoperative adjuvant chemotherapy. Her high cancer antigen 125 (CA125) serum level of 79 U/mL before radical hysterectomy was considered to mainly originate from the endometrial cyst of the left ovary, and this level decreased to 5.1 U/mL after postoperative adjuvant chemotherapy. Three years after the primary treatment, the patient’s CA125 serum level was elevated at 161 U/mL, and it further increased to 1260 U/mL 10 months later (Fig. 1). She began to feel chest pain, and computed tomography showed swelling of left supraclavicular lymph nodes and mediastinal lymph nodes (Fig. 2). Biopsy of a left supraclavicular lymph node showed metastasis of squamous cell carcinoma, and type 18 human papillomavirus DNAs were detected by molecular biological analysis. She was a nonsmoker, and we considered the presence of human papilloma virus deoxyribonucleic acid (DNA) showed that the lymph node metastasis originated from cervical cancer; the endoscopic examination for upper digestive tract was not scheduled. The patient underwent chemoradiotherapy at 60 Gy/30 times for 6 weeks combined with an intravenous cisplatin drip weekly at 40 mg/m^2^.

Three weeks after beginning chemoradiotherapy, the patient’s chest pain decreased in severity. Six weeks after beginning chemoradiotherapy, the left supraclavicular lymph node swelling disappeared, and computed tomography showed that the mediastinal lymph nodes had decreased in size. The serum level of CA125 decreased to 175 U/mL. The serum level of SCCA was 1.2 ng/mL before chemoradiotherapy and 0.9 ng/mL after chemoradiotherapy. After the chemoradiotherapy, the patient received four cycles of chemotherapy with irinotecan and cisplatin followed by six cycles of chemotherapy with paclitaxel and carboplatin. The serum levels of CA125 and SCCA were then measured at 12.8 U/mL and < 0.5 ng/mL, respectively. At the time of this writing, the patient had been followed in an outpatient clinic for more than 8 years, and no further metastasis has been found.

**Fig. 3:**
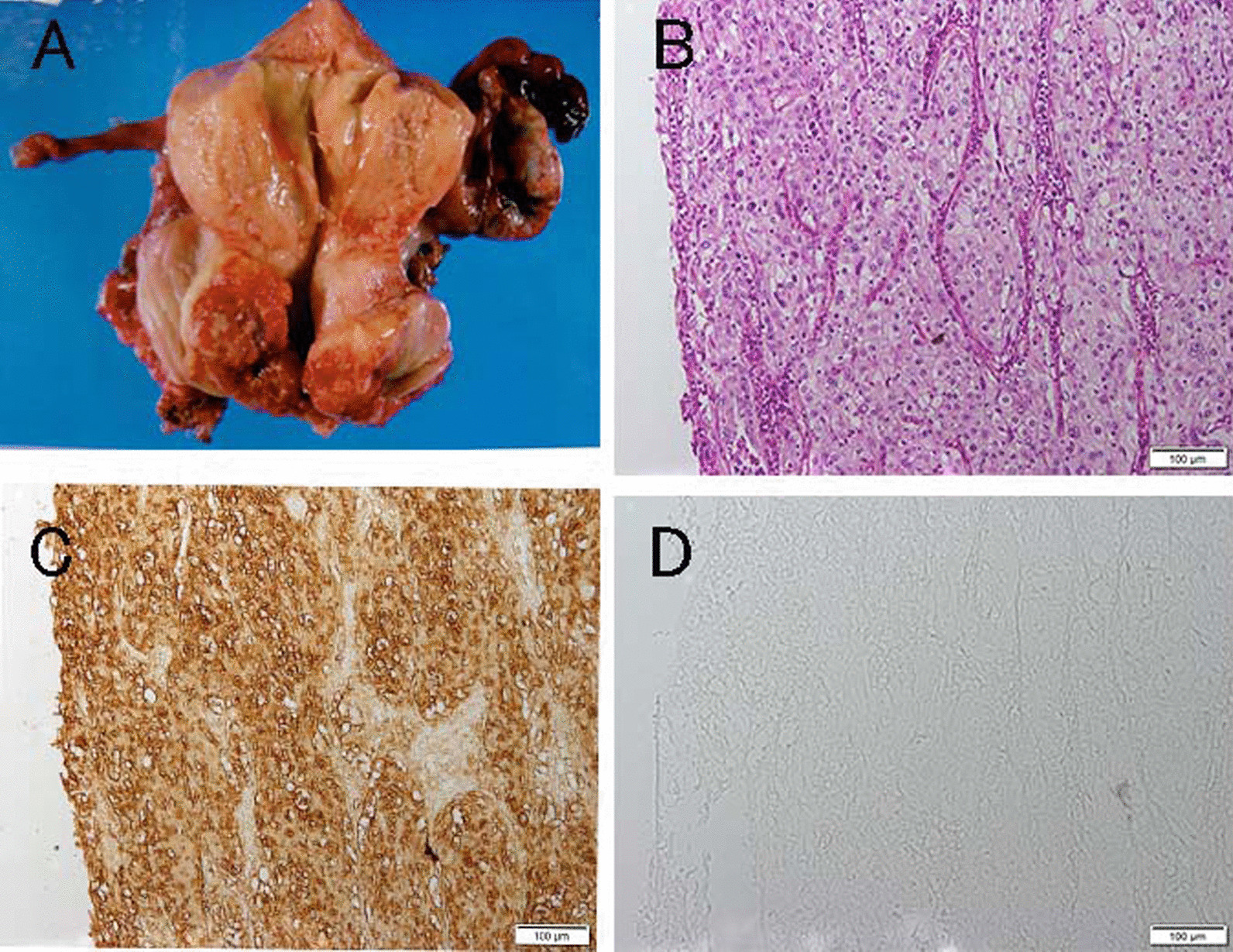
Examination of affected tissues. **A** Macroscopic specimens of uterus and left adnexa. **B**–**D** Cervical cancer tissue. **B** Hematoxylin–eosin staining. **C** Immunohistology with SCCA1/SCCA2 antiserum (CAC, Cosmo Bio SU-IZ-PO4). **D** Immunohistology with anti-CA125 mouse monoclonal antibody (CMC, 325M-17)

**Fig. 4:**
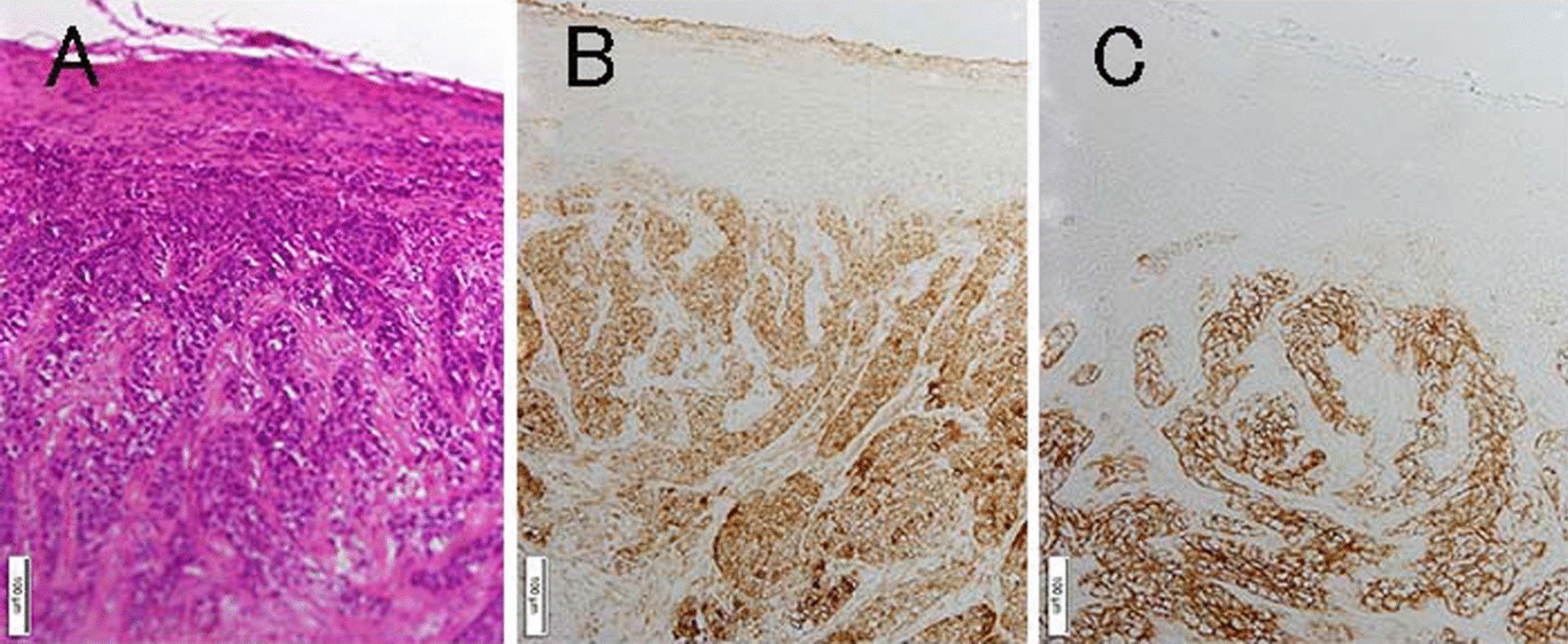
Examination of supraclavicular lymph nodes. **A** Hematoxylin–eosin stain. **B** Immunohistology with SCCA1/SCCA2 antiserum (CAC, Cosmo Bio SU-IZ-PO4). **C** Immunohistology with anti-CA125 mouse monoclonal antibody (CMC, 325M-17)

We considered that the elevated serum CA125 level originated from metastatic tumor tissue in the lymph nodes. We therefore immunohistochemically analyzed the tissues of the original cervical cancer and supraclavicular lymph nodes with antibodies to SCCA and CA125. Anti-SCCA1/SCCA2 antiserum showed cancer cells in the original cervical tumor (Fig. 3) and metastatic cancer cells in the supraclavicular lymph nodes (Fig. 4). Anti-CA125 monoclonal antibody showed metastatic cancer cells in the lymph nodes but no cancer cells in the original cervical cancer tissues.

## Discussion and conclusion

Biomarkers are utilized to identify the occurrence and recurrence of neoplasia. SCCA is the major biomarker for squamous cell carcinoma of the cervix or other organs that contain squamous cells [7]. After the first treatment of the neoplasm, the biomarker for recurrence is usually the same as that for the first diagnosis. In this patient, the level of the biomarker CA125 was elevated at the time of postoperative mediastinal and supraclavicular lymph node metastasis despite the fact that there was no change in the level of SCCA (the biomarker of the original cervical cancer).

CA125 is a target of a monoclonal antibody raised against the epithelial ovarian cancer cell line OVCA433. CA125 led to elucidation of a peptide epitope of MUC16, a high-molecular-weight mucin spanning the cell membrane. Research has shown that CA125 expressed on ovarian cancer cells protects these cells from natural killer cells and monocytes [8, 9]. Other research has shown that high expression of CA125 on ovarian cancer cells triggers peritoneal metastasis of these cells [10]. There are also some reports that serum CA125 is elevated in patients of lung cancer and other thoracic diseases that are accompanied by pleural effusion [11].

It is shown that CA125 in cell membrane protects cells from attack of natural killer cells and monocytes. Other research suggests that CA125 plays a role in peritoneal metastasis of cells by an interaction of *N*-glycans to mesothelial lining [10]. E-cadherin-mediated cell invasion and migration are also induced by an interaction of cytoplasmic tail of CA125 with Src-family kinase [12]. In breast cancer cells, Src-family kinase is shown to be related with latent bone metastasis [13]. In endometrial cancer, elevated serum CA125 is shown in patients with pelvic or para-aortic lymph node metastasis [14]. Although the serum CA125 level is not markedly elevated in many patients who have cervical cancer with lymph node metastasis, there is a report showing combined scores of SCC and CA125 in serum are related to metastasis in pelvic or para-aortic lymph nodes [15]. In cervical cancer, metastasis to the mediastinal and supraclavicular lymph nodes, such as in our experience, may be related to factors different from those to the pelvic and para-aortic lymph nodes and reflected on serum CA125.

## Data Availability

All data presented in this report are included in this article.
